# Effects of wollastonite and phosphate treatments on cadmium bioaccessibility in pak choi (*Brassica rapa* L. ssp. *chinensis*) grown in contaminated soils

**DOI:** 10.3389/fnut.2024.1337996

**Published:** 2024-04-04

**Authors:** Kexin Guo, Yuehua Zhao, Yang Zhang, Jinbo Yang, Zhiyuan Chu, Qiang Zhang, Wenwei Xiao, Bin Huang, Tianyuan Li

**Affiliations:** ^1^Shandong Provincial Key Laboratory of Applied Microbiology, Ecology Institute, Qilu University of Technology (Shandong Academy of Sciences), Ji’nan, China; ^2^The 7th Institute of Geology & Mineral Exploration of Shandong Province, Linyi, China; ^3^Weifang Binhai Ecological Environment Monitoring Center, Weifang, China; ^4^Guangzhou Hexin Instrument Co., Ltd., Guangzhou, China; ^5^Zhongchuang Guoke Scientific Instrument (Shandong) Co., Ji’nan, China

**Keywords:** Cd immobilization, bioaccessibility, leafy vegetables, wollastonite, cell ultrastructural

## Abstract

Cadmium (Cd) contamination of soil can strongly impact human health through the food chain due to uptake by crop plants. Inorganic immobilizing agents such as silicates and phosphates have been shown to effectively reduce Cd transfer from the soil to cereal crops. However, the effects of such agents on total Cd and its bioaccessibility in leafy vegetables are not yet known. Pak choi (*Brassica rapa* L. ssp. *chinensis*) was here selected as a representative leafy vegetable to be tested in pots to reveal the effects of silicate–phosphate amendments on soil Cd chemical fractions, total plant Cd levels, and plant bioaccessibility. The collected Cd contaminated soil was mixed with control soil at 1:0, 1:1, 1:4, 0:1 with a view to Cd high/moderate/mild/control soil samples. Three heavy metal-immobilizing agents: wollastonite (W), potassium tripolyphosphate (KTPP), and sodium hexametaphosphate (SHMP) were added to the soil in order to get four different treatment groups, i.e., control (CK), application of wollastonite alone (W), wollastonite co-applied with KTPP (WKTPP), application of wollastonite co-applied with SHMP (WSHMP) for remediation of soils with different levels of Cd contamination. All three treatments increased the effective bio-Cd concentration in the soils with varying levels of contamination, except for W under moderate and heavy Cd contamination. The total Cd concentration in pak choi plants grown in mildly Cd-contaminated soil was elevated by 86.2% after WKTPP treatment compared to the control treatment could function as a phytoremediation aid for mildly Cd-contaminated soil. Using an *in vitro* digestion method (physiologically based extraction test) combined with transmission electron microscopy, silicate and phosphorus agents were found to reduce the bioaccessibility of Cd in pak choi by up to 66.13% with WSHMP treatment. Application of silicate alone reduced soil bio-Cd concentration through the formation of insoluble complexes and silanol groups with Cd, but the addition of phosphate may have facilitated Cd translocation into pak choi by first co-precipitating with Ca in wollastonite while simultaneously altering soil pH. Meanwhile, wollastonite and phosphate treatments may cause Cd to be firmly enclosed in the cell wall in an insoluble form, reducing its translocation to edible parts and decreasing the bioaccessibility of Cd in pak choi. This study contributes to the mitigation of Cd bioaccessibility in pak choi by reducing soil Cd concentration through *in situ* remediation and will help us to extend the effects of wollastonite and phosphate on Cd bioaccessibility to other common vegetables. Therefore, this study thus reveals effective strategies for the remediation of soil Cd and the reduction of Cd bioaccessibility in crops based on two indicators: total Cd and Cd bioaccessibility. Our findings contribute to the development of methods for safer cultivation of commonly consumed leafy vegetables and for soil remediation.

## 1 Introduction

Soil contamination with cadmium (Cd) has become a major environmental and public health challenge in countries such as China, Thailand, and India (1, 2). Cd readily migrates from the soil into crop plants, thus entering the food chain and posing a risk to human health. For example, Chinese cabbage plants grown in a specific region of China reportedly have maximum Cd contents of 0.09 mg/kg, exceeding the standard limit by 180% (3). Cd has also been found to exceed the standard by 53.85% in vegetables from Yunnan Province (4). Thus, effective methods are urgently needed to control Cd contamination to limit Cd transfer through the food chain.

The health risks associated with heavy metal elements in crops can be reduced through two key strategies. The first is to decrease the total concentration of heavy metals taken up by crop plants. In prior studies, immobilizing agents have proven effective in reducing heavy metal uptake from the soil (5, 6). Common heavy metal immobilizing agents include organic materials, such as compost and biochar; inorganic materials, including lime, phosphate, and silicate; and chemical chelating agents, such as ethylenediaminetetraacetic acid (EDTA) and diethylenetriaminepentaacetic acid (DTPA) (7). For example, the addition of bagasse biochar to paddy soil significantly reduced the DTPA extractable fraction of Cd and reduced the Cd content of rice plants (8). Another study found that biochar reduced Cd accumulation in pak choi (9). Similarly, straw composting resulted in a significant 69% reduction in Cd content in pak choi, as well as a reduced Cd transfer factor from soil to pak choi leaves (10). One promising inorganic immobilizing material is a calcium silicate material, wollastonite. This compound combines with heavy metals to form Si-Cd precipitates, which are less readily absorbed by plants than free Cd. Several studies have demonstrated the efficacy of wollastonite in reducing heavy metal mobility and toxicity (11–14). Phosphate is another common heavy metal immobilizing treatment for soils. The addition of phosphorus reduces the concentration of Cd (water-soluble and exchangeable) released into the soil solution in the soil, reducing the migration of its Cd to plants (15). Field experiments have demonstrated that it effectively reduces Cd concentrations in rice (16). In addition, co-application of silicate and potassium dihydrogen phosphate enhances soil adsorption of Cd, minimizing Cd uptake by altering the proportions of competing cation fractions in the soil. This has been shown to reduce Cd concentrations in amaranth and Chinese cabbage by up to 74% (17). Recently, Wang et al. (18) found that a combined application of silicate and phosphate decreases the exchangeable Cd concentration in the soil by altering the soil pH or causing direct Cd adsorption to the surfaces of silicate minerals. Such prior studies have identified strong candidates for Cd remediation via immobilization in the soil. However, most of the current studies are on the effect of single immobilizers on Cd in vegetables or other crops, and the effect of wollastonite and phosphate on Cd in leafy vegetables, especially pak choi, is not yet known, and further studies are needed to clarify the potential mechanisms of the effect.

The second method of mitigating health risks associated with heavy metal contamination is to reduce their bioaccessibility in plants. Bioaccessibility refers to the proportion or amount of a compound in a food product that can be digested and extracted in the gastrointestinal environment. In the context of heavy metals, the total contaminant mass within a crop may not accurately reflect the mass that can be absorbed by the human body (19). Research has indicated that bioaccessibility is a more accurate reflection of human absorption and utilization of heavy metals than total heavy metal concentrations are. Thus, it is important to understand heavy metal bioaccessibility in crops. For example, Wang et al. (20) found that Cd bioaccessibility in peppermint can reach 85.8%, posing a significant health risk to humans. Similarly, Hu et al. (21) found that Cd bioaccessibility in leafy vegetables from market in Hong Kong is 71%, making it a major health risk for residents. Prior studies have indicated that Cd contamination poses a major health risk due to its generally high bioaccessibility. Strategies for minimizing bioaccessibility Cd are thus urgently needed.

In addition to preventing plant uptake of heavy metals, immobilizing agents may also impact bioaccessibility. For example, biochar can transform the exchangeable and carbonate-bound fractions of Cd into organic-bound and residual fractions. In wheat and corn, this reduces the bioaccessible Cd content by 12.7–26.0% and 13.1–20.5%, respectively (22). In celery production, a combined application of hydroxyapatite with slaked lime or hydroxyapatite with zeolite produce synergistic effects, significantly increasing soil pH and reducing Cd bioaccessibility by 54.8–79.0% (23). These types of immobilizing agents may therefore reduce heavy metal bioaccessibility in other crop plants as well. However, to date, little is known about the bioaccesssibility of Cd in pak choi harvested from agricultural soils remediated by *in situ* soil immobilization of Cd and the associated risks associated with its consumption. The beneficial effects of *in situ* soil Cd immobilization in reducing Cd accumulation in other crops such as rice have been well documented (24, 25). However, its effect on the bioaccessibility of Cd in vegetables has not received sufficient attention.

Bioaccessibility content of heavy metals in agricultural products is a key indicator for assessing health risks. And chard is more likely to absorb Cd when grown on contaminated soils, leading to an increase in potential health risk (26). The addition of passivator will immobilize Cd more in the soil and reduce its migration to pak choi, meanwhile, wollastonite has non-specific immobilization and can immobilize some trace elements in the soil, and the addition of phosphorus will also change the soil nutrients. Passivator combinations of wollastonite and phosphate have been used to reduce the risk of Cd exposure in paddy and rice, however, their effect on the bioaccessibility of Cd in vegetables remains unclear. Therefore, there is a need to use a combination of wollastonite and phosphate to reduce the total amount and bioaccessibility of cadmium in the edible parts of aubergines. Prior studies of Cd bioaccessibility have primarily focused on the effects of immobilizing agents on total heavy metal concentrations in crops such as grains; research on the bioaccessibility of Cd among leafy vegetables grown in contaminated soils is extremely scarce. However, recent dietary trends have emphasized consumption of leafy green vegetables over grains, increasing the overall health risks posed by heavy metal contamination of leafy vegetables. Thus, there is a pressing need to assess Cd bioaccessibility in such plants. To address this goal, the present study had three key aims: (1) to investigate the effects of a combination of silicate and different phosphates on total Cd concentrations in the leafy green vegetable pak choi (*Brassica rapa* L. ssp. *chinensis*); (2) to assess changes in Cd chemical fractions in the soil after application of silicate and phosphates; and (3) to study the effects of a silicate–phosphate combination on Cd bioaccessibility and toxicity in pak choi. This study was designed to aid in determining appropriate strategies for controlling Cd exposure in specific populations, reducing the health risks posed by heavy metal exposure in the context of modern dietary patterns.

## 2 Materials and methods

### 2.1 Soil collection

Soil samples contaminated with Cd were collected from 0 to 20 cm topsoil of a barren land outside a chemical industrial park in Linyi City, Shandong Province, China (Supplementary Figure S1). Control soil samples were simultaneously collected ~5 km away from the industrial park. The basic properties of the experimental soil were analyzed before experimenting. Soil pH and conductivity were measured with a pH meter and a conductivity meter at a ratio of 1:2.5 soil to distilled water. Soil organic matter was measured by potassium dichromate method by oxidising soil organic carbon with excess potassium dichromate-sulphuric acid solution under heated conditions and excess potassium dichromate was titrated with ferrous sulphate standard solution. The dry matter content was obtained by drying and weighing the difference, and the basic physio-chemical properties of the collected soils were as follows (27). The pH of the Cd-contaminated soil was 7.297, with 1.94% organic matter and 97.0% dry matter content. The pH of the control soil was 7.316, with 1.99% organic matter and 97.1% dry matter content (Table 1).

**Table 1:** Physical and chemical properties of soil before and after treatment.

Properties	Values	
	Before treatment	After treatment
pH	7.29–7.32	8.15–8.65
Organic matter content (%)	1.99	1.91
Dry matter content (%)	97.0	97.6
Cation exchange capacity (CEC, cmol kg^−1^)	13.57	13.16
Conductivity (μs cm^−1^)	128.5	126.4
Texture	Brown loam	

### 2.2 Determination of soil Cd concentration

For each sample, 15 mL of aqua regia was transferred to a conical 100 mL flask that had been infiltrated by aqua regia vapors. An additional 6 mL of aqua regia solution was added and a glass funnel was placed at the top. Each flask was heated on an electric hot plate to maintain the aqua regia at a slight boiling state for 2 h. Then, samples were cooled to room temperature and allowed to settle. The extract was then slowly filtered through quantitative filter paper into a 50 mL volumetric flask. The glass funnel, conical flask, and residue were rinsed with a small amount of nitric acid solution at least three times, with the reinstate collected in the volumetric flask. After filtration, the inductively coupled plasma mass spectrometry (ICP-MS 1600, Hexin Mass Spectrometry, China) was used to determine Cd concentrations in the digested sample solutions.

### 2.3 Pot-planting experiment

The Cd-contaminated and control soils were mixed at ratios of 1:0, 1:1, 1:4, and 0:1 to yield soil samples with a range of Cd contamination levels: heavy contamination (6.28 mg/kg), moderate contamination (3.21 mg/kg), mild contamination (1.36 mg/kg), and uncontaminated soil (0.14 mg/kg). Several compounds were then tested to assess the Cd-immobilizing effects in each soil type. Wollastonite (a low cost calcium silicate mineral) was used as the main immobilizing agent of the crop and two different phosphate crops, sodium tripolyphosphate (KTPP) and sodium hexametaphosphate (SHMP), were selected to enhance the immobilizing effect of Cd based on the results of Matusik et al. and Thawornchaisit and Polprasert (28, 29). The silica fume and phosphate salt dosages were 400 mg Si kg^−1^ soil and 0.4% P_2_O_5_ kg^−1^ soil, respectively. The treatments consisted of a control check (CK), wollastonite (W), wollastonite with potassium triphosphate (WKTPP), and wollastonite with sodium hexametaphosphate (WSHMP). Each treatment was applied to each of the four soil types with differing Cd contamination levels in biological triplicate. After thoroughly mixing wollastonite and phosphate with the soil samples were incubated for 14 d soil moisture was maintained at ~70% of the field capacity. Treated soil samples were air-dried, ground, and sieved through 2 mm mesh. Each pot was filled with 0.75 kg of soil. Fertilizers were applied to all pots in the forms of urea and KCl to final concentrations of 0.2 g N and 0.15 g K per kg of soil, respectively. In the blank and W treatment groups, Na_2_HPO_4_ was added to a final concentration of 0.4% P_2_O_5_ per kg of soil.

Pak choi seeds were purchased from Sanjiang Agriculture (Zhejiang Province, China) (30) and three to four seeds were planted in each pot. After the seedlings grew four true leaves, they were thinned to one plant per pot, and all potted plants were placed in a 24 h light incubator, with the temperature maintained at 23°C and humidity at about 60%. After 45 d, the aboveground plant portions were harvested rinsed with tap water, then thoroughly washed with ultrapure water. After removing excess surface moisture, samples were weighed to determine the fresh weight. A portion of each sample was finely chopped with a ceramic knife, mixed well, and stored in a refrigerator at 4°C prior to additional analyses. The remaining portions was dried in an oven at 70°C to constant weight for further analysis.

### 2.4 Determination of soil Cd chemical fractions

Nowadays, methods such as BCR and Tessier are mostly used for the determination of heavy metal forms, and in this study, soil samples were analyzed using an improvement upon the Tessier sequential extraction method (31), the Tessier seven-step extraction method (32). Elements were divided into different forms: water-soluble (F1), ion exchangeable (F2), carbonate-bound (F3), humic acid-bound (F4), iron-manganese oxide-bound (F5), strong organic-bound (F6), and residual forms (F7). F1, F2, and F3 represent bioaccessible Cd (bio-Cd), which has better mobility and migration in soil and can be bioaccessible by plants, whereas F4–F7 represent inert Cd (inert-Cd), which is not easy to migrate in soil and is more stable. The specific analysis method is shown in Table 2.

**Table 2:** Tessier’s seven-step sequential extraction method for Cd in soil.

Step	Fraction	Reagent(s)	Experimental conditions
I	Water-soluble	Distilled water	2.5 g soil sample reagents. Echocardiography: 30 min
II	Ionic bond	1 M magnesium chloride	Residual fraction +25 mL reagents. Echocardiography: 30 min
III	Carbonate	1 M NaAc	Residual fraction +25 mL reagents. Echocardiography: 1 h
IV	Humic acid-bound	0.1 M Na_4_P_2_O_7_	Residual fraction +50 mL reagents. Echocardiography: 40 min
V	Fe/Mn oxide bound	0.25 M NH_2_OH•HCl–HCl	Residual fraction +50 mL reagents. Echocardiography: 1 h
VI	Strong organic	0.02 M HNO_3_ H_2_O_2_ (pH 2)	Residual fraction +3 mL HNO_3_ + 5 mL H_2_O_2_ 83°C constant temperature: 1.5 h
		3.2 M NH_4_Ac-HNO_3_	Residual fraction +2.5 mL NH_4_Ac-HNO_3_ Set aside: 10 h Centrifugation: 20 min
VII	Residual	Ultrapure water	Residual fraction +20 mL reagents. Oscillate: 15 min

### 2.5 Determination of Cd concentrations in pak choi

Dry samples (0.5 g) were placed in tetrafluoroethylene inner jars. After adding 5 mL of nitric acid, samples were soaked overnight, then 2–3 mL of hydrogen peroxide solution was added. After adding 2–3 mL of 30% hydrogen peroxide solution, the inner lid was covered, the stainless-steel jacket was tightened, and jars were incubated in a drying oven at 120–160°C for 4–6 h. Samples were naturally cooled to room temperature in the incubator, then opened and heated until nearly dry. The digestive solution was washed into a 25 mL volumetric flask before the inner jar and inner lid were washed with a small amount of 1% nitric acid solution three times. The rinsates were combined in a volumetric flask and fixed to 25 mL scale with 1% nitric acid solution. Samples were mixed; at the same time, repeat the above procedure without adding samples as a blank control. The Cd contents in the digested sample solutions were then determined with an ICP-MS instrument (ICP-MS 1600, Hexi Mass Spectrometry, China). The main instrumental operating parameters for the determination of the samples are shown in Table 3. After ignition of the plasma, the ICP-MS was preheated for 30 min, and the sensitivity, oxide and double charge of the instrument were tuned with 1.0 μg/L tuning solution, and the relative standard deviation of the signal intensity of the elements contained in the tuning solution was less than or equal to 0.8% under the conditions that the sensitivity, oxide and double charge of the instrument met the requirements. The results showed that the precision RSD ranged from 0.5 to 12.3%; the recoveries of the certified standard samples ranged from 86.4 to 114.3%, and the limits of detection, precision and correctness were all in accordance with the standard determination requirements.

**Table 3:** The major instrumental operation parameters for soil and food samples determination.

Instrumental operation parameters		Values
Radio frequency power of plasma		1,400 W
Depth of sampling		9.70 mm
Extraction voltage		−910 V
Gas flow rates	Carrier gas	0.80 L/min
	Dilution gas	0.30 L/min
	Collision gas	1.48 mL/min

### 2.6 Determination of the Cd bioaccessibility in pak choi

Currently, PBET is used as an *in vitro* method to determine the bioaccessibility of Cd in the literature. Therefore, an improved method based on those described by experimental methods of Ruby et al. (33) and Fu et al. (34) was employed to determine the bioaccessibility of Cd in aboveground pak choi tissues using the Physiologically Based Extraction Test (PBET) method. In the simulated gastric digestion stage, 3 g of fresh sample was mixed with 30 mL of simulated gastric fluid containing 0.50 g/L sodium citrate, 0.50 g/L sodium malate, 0.42 mL/L lactic acid, 0.50 mL/L acetic acid, and 1.25 g/L gastric protease (pH adjusted to 1.50 using concentrated hydrochloric acid). The mixture was then rotated and oscillated at 4000 rpm and 37°C for 1 h. After centrifugation at 4000 rpm or 10 min, 5 mL of the supernatant was filtered and collected for further testing. In the simulated intestinal digestion stage, 5 mL of gastric fluid was added to the reaction solution to maintain a constant solid: liquid ratio. The pH of the digestion solution was adjusted to 7.0 with solid sodium bicarbonate (NaHCO_3_). Bile salt solution was prepared by diluting 52.5 g/L porcine bile salt solution with 0.1 mol/L NaHCO_3_ solution, then adding 15 mg of pancreatin. Each sample was combined with 1 mL of the bile salt solution, then the mixture was rotated and oscillated at 4000 rpm and 37°C for 4 h. After centrifugation at 4000 rpm for 10 min, the supernatant was collected and the precise volume recorded. The digestion solution of the small intestine phase was filtered through a 0.45 μm filter membrane, and the Cd content was determined by ICP-MS (ICP-MS 1600, Hexi Mass Spectrometry, China) together with the gastric digestion solution.

The bioaccessibility of cadmium was assessed during the gastric and intestinal digestion stages of cabbage samples. Equation (1) was used for calculation (35).


(1) $$BAC\;\left(\%\right)=\frac{C1V1}{C2M}\times 100\%$$

where BAC is the percent bioaccessibility of Cd in aboveground plant tissue in the gastric or small intestinal stage; C1 is the Cd content in the reaction solution at that stage (μg·mL^−1^); V1 is the volume of the reaction solution at that stage (mL); C2 is the Cd content in the aboveground plant tissue (μg·g^−1^ DW); and M is the original mass of the sample used in the simulated digestion experiment (g).

### 2.7 Transmission electron microscopy

The second developed leaves were collected from pak choi plants grown for 35d. Slices of 2 mm^2^ in size were taken from the tops of the leaves in the subapical region (without veins or root tips). The slices were set for 12 h with 2.5% glutaraldehyde (v/v) in 0.1 M phosphate-buffered saline (PBS) at pH 7.0. After washing twice in PBS, samples were post-fixed in 1% (v/w) OsO_4_ for 2 h, then washed three times with 0.1 M PBS 0.1 M for 10 min each. The samples were then dehydrated in an ethanol gradient (50, 60, 70, 80, 90, 95, and 100%) with 15–20 min between solutions before washing for 20 min in absolute acetone. Prepared samples were fixed in Spurr’s resin overnight, then heated at 70°C for 9 h. Ultrathin (80 nm) sections of the samples were set up under an accelerating voltage of 60.0 kV, mounted on a copper grid, and observed under TEM (Hitachi-7800). The magnification of 5,000 and 30,000 was chosen to observe the overall cellular and subcellular changes. Images that best represented the ultrastructural cell states were selected for analysis.

### 2.8 Transfer factor

The TF of Cd from the soil to the edible portions of pak choi was defined as the ratio of the Cd concentration in the edible parts of the vegetable (in mg kg^−1^) over the total cadmium concentration in the planting soil (mg kg^−1^) (36, 37) and was calculated using Equation 2.


(2) TF=Cplant/Csoil

Where Cplant and Csoil represents the Cd concentration in plants and soils on dry weight basis, respectively.

### 2.9 Statistical analysis

SPSS (SPSS Statistics 25) was used for statistical analysis. Data were analyzed by one-way ANOVA with Duncan’s New Multiple Range Test to determine significant differences between different. Pearson’s correlation test was used to establish the correlation between Cd bioavailability and total Cd in *Brassica napus* and between total pak choi Cd and total soil Cd at a significant level of *p* < 0.05 (two-tailed). Cd concentration and chemical form in soil and pak choi are given as means and standard errors, *p* < 0.05, and were analyzed by one-way ANOVA and post-hoc Tukey’s honestly significant difference test because of the grouped observed variables. All of the data presented are means ± SE of three replicates. All graphs were plotted using Origin 2022.

## 3 Results and discussion

### 3.1 Cd soil chemical fractions

**Figure 1:**
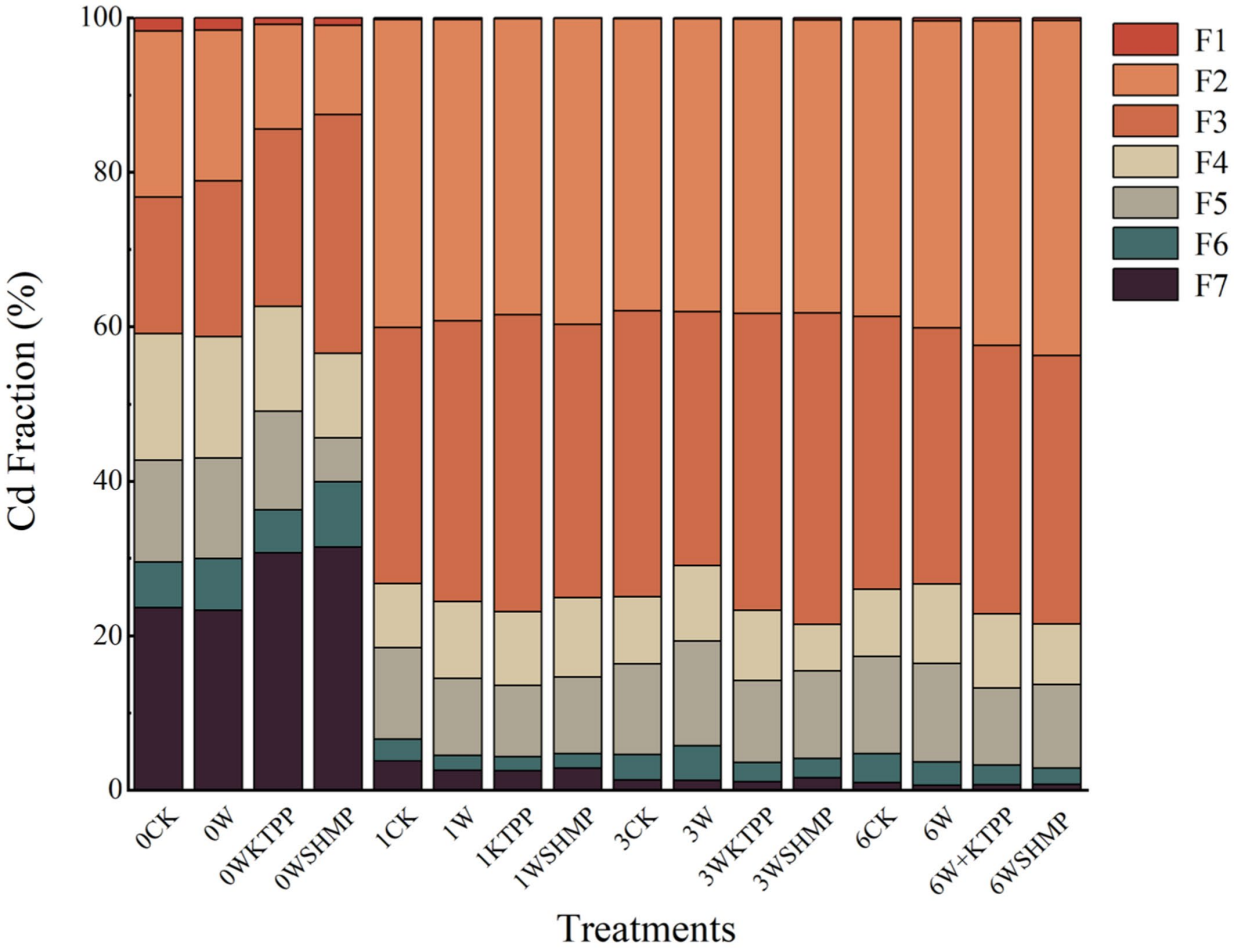
Cd chemical fractions in soils with varying degrees of Cd contamination in response to treatment with immobilizing agents. F1, water-soluble fraction; F2, ionic bond fraction; F3, carbonate fraction; F4, humic acid-bound fraction; F5, Fe/Mn oxide-bound fraction; F6, strong organic fraction; F7, residual fraction. CK, untreated control soil; W, wollastonite treatment; WKTPP, wollastonite + potassium triphosphate treatment; WSHMP, wollastonite + sodium hexametaphosphate treatment. Numbers before treatment group names indicate the soil Cd abundance: 0, control soil; 1, mild Cd contamination; 3, moderate Cd contamination; 6, high Cd contamination.

We first assessed the impacts of three immobilizing treatments, W, WKTPP and WSHMP on soil properties. All three treatments increased the soil pH from 7.29–7.32 to 8.15–8.65. Furthermore, the bioaccessible Cd fractions (bio-Cd) (F1–F3) were more abundant than inert Cd fractions (inert-Cd) (F4–F8) after each treatment (Figure 1). In mildly Cd-contaminated soil, all three treatments reduced the proportion of inert-Cd; the proportion of bio-Cd in the WKTPP treatment group reached 76.8%, which was 20.48% higher than in the untreated control soil. In moderately Cd-contaminated soil, inert-Cd was reduced by WKTPP and WSHMP treatment, in which the bio-Cd proportions reached 76.7 and 78.5%, respectively. However, W treatment reduced the bio-Cd percentage from 75 to 70.9%. In highly Cd-contaminated soil, alterations in inert-Cd and bio-Cd contents were consistent with those in moderately Cd-contaminated soil, with WKTPP and WSHMP treatment increasing the bio-Cd proportions by 3.2 and 4.5%, respectively.

In general, application of W alone reduced bio-Cd levels in the tested soils, whereas combined wollastonite–phosphate applications increased bio-Cd concentrations. Soil Si has previously been shown to adsorb Cd to form silicate complexes. The resulting complexes have a limited capacity for ion exchange, limiting their solubility in soil water (38, 39). This is the reason for the decrease in bio-Cd in the soil under the W treatment alone in Figure 1.

Furthermore, the mineral form of Si has silanol (Si-OH) groups that can adsorb Cd, decreasing Cd mobility because it is kept in the stable Cd-Si-OH form (40). Unstable forms of Cd also bind to Si through oxygen bridges to create insoluble Fe-O-Si-Cd complexes (41). This is in line with the finding of Feng et al. that Si is able to transform heavy metal fractions in soil by producing Si complexes, thus reducing bioaccessible Cd (42). Phosphate alone can bind with Cd to form metal–phosphate precipitates, thus reducing bio-Cd content. However, wollastonite is Ca-based; in a simultaneous application of wollastonite with a phosphate, the phosphate will form precipitates with Ca (43), reducing binding of both compounds to soil Cd and thus increasing soil bio-Cd concentrations. Furthermore, the elevated pH resulting from treatment with wollastonite and phosphate decreases the bioaccessibility of iron (Fe) and manganese (Mn). Because Cd competes for metal transporters with Fe and Mn, this treatment results in increased competitive adsorption and bioaccessibility of Cd^2+^, promoting transportation into the soil–plant system (44, 45). Thus in Figure 1 it can be seen that the simultaneous application of wollastonite and phosphate instead led to an increase in bio-Cd concentration in the soil.

Little attention has been given in previous studies to the effects of combinations of passivators on soil Cd fractions, and this study found that Si and P in wollastonite and phosphate will combine with Cd in the soil to form precipitates and complexes to reduce the bio-Cd content. However, the addition of wollastonite and phosphate at the same time reduces the precipitation and complexation with Cd because Ca is the first to bind to P, resulting in an increase in bio-Cd content of soil in most of the treatment groups, especially in the WSHMP treatment group in moderately contaminated soil. Therefore, the simultaneous addition of immobilizing agents combination containing Si material and phosphate will instead promote the conversion of soil Cd to plant bioaccessible fractions.

### 3.2 Total Cd concentrations in aboveground pak choi tissues

**Figure 2:**
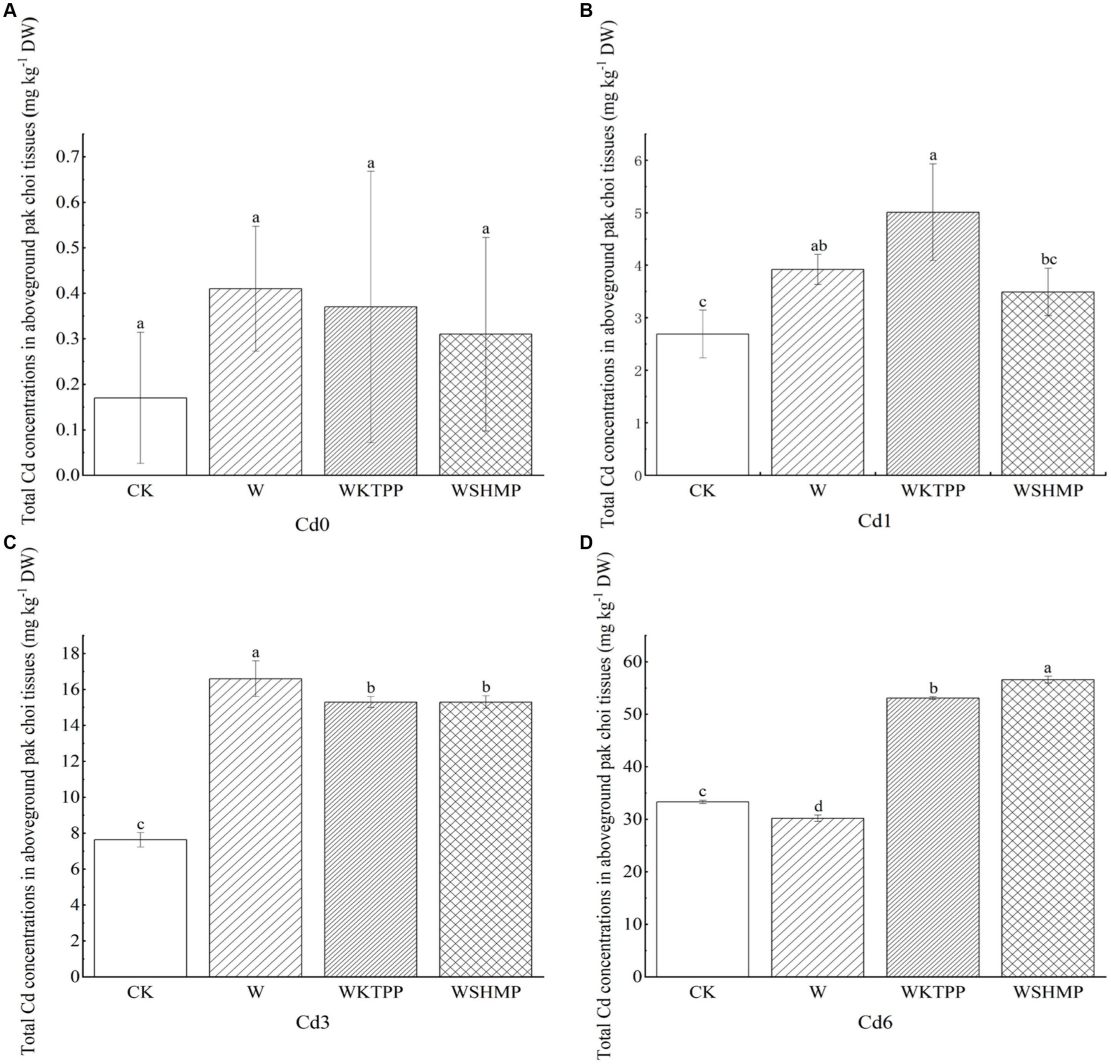
Total Cd concentrations in aboveground pak choi tissues in **(A)** Cd0, **(B)** Cd1, **(C)** Cd3, and **(D)** Cd6 soils after treatment with immobilizing agents. CK, untreated control soil; W, wollastonite treatment; WKTPP, wollastonite with potassium triphosphate treatment; WSHMP, wollastonite with sodium hexametaphosphate treatment. Numbers before treatment group names indicate the soil Cd abundance: 0, control soil; 1, mild Cd contamination; 3, moderate Cd contamination; 6, high Cd contamination. Letters above each bar indicate statistical significance groups at *p* < 0.05 (one-way analysis of variance with post-hoc Tukey’s honestly significant difference test).

We next assessed the effects of each treatment on Cd concentrations in aboveground pak choi tissues. In highly Cd-contaminated soil, W treatment decreased the total aboveground Cd concentration. All other treatment group–soil combinations showed increased total aboveground Cd levels (Figure 2). For example, in moderately Cd-contaminated soil, W treatment significantly increased the total Cd in aboveground tissues to 16.61 mg/kg from 7.63 mg/kg in the control. In mildly and highly Cd-contaminated soil, Cd uptake by the plant was highest in the combined W + phosphate treatments. Specifically, WKTPP treatment increased the total aboveground pak choi Cd contents by 86.2 and 59.5% in mildly and highly Cd-contaminated soils, respectively; WSHMP treatment increased the Cd contents among plants grown in the same soils by 29.7 and 69.9%, respectively (Table 4). In mildly Cd-contaminated soils, the Cd transfer factor (TF) increased by 71.6% in the WKTPP treatment group compared to the control. In moderately and highly Cd-contaminated soils, WSHMP treatment increased the TF by 74.4 and 51.0%, respectively (Supplementary Tables S1, S3). This is generally consistent with changes in Cd fractions in the soil.

**Table 4:** Effects of *in situ* soil treatment with immobilizing agents on total Cd mass and Cd bioaccessibility in pak choi.

Soil treatment group	Total Cd (mg kg^−1^)	Cd Bioaccessibility (%)	
		Gastric	Intestinal
0CK	0.17 ± 0.14	122.49 ± 52.72	16.03 ± 5.96
0 W	0.41 ± 0.13	94.54 ± 24.43	13.78 ± 0.06
0 WKTPP	0.37 ± 0.30	71.96 ± 10.06	9.73 ± 0.35
0 WSHMP	0.31 ± 0.21	111.60 ± 21.99	12.71 ± 1.73
1CK	2.69 ± 0.45	100.41 ± 8.78	9.82 ± 0.84
1 W	3.92 ± 0.28	57.42 ± 3.62	6.03 ± 0.85
1 WKTPP	5.01 ± 0.92	49.52 ± 0.82	5.59 ± 0.36
1 WSHMP	3.49 ± 0.45	92.01 ± 5.88	12.60 ± 3.10
3CK	7.63 ± 0.40	56.87 ± 21.83	8.73 ± 0.38
3 W	16.62 ± 0.99	45.15 ± 5.21	5.54 ± 0.75
3 WKTPP	15.31 ± 0.30	78.96 ± 6.22	10.27 ± 1.05
3 WSHMP	15.25 ± 0.35	40.37 ± 14.13	5.98 ± 1.57
6CK	33.27 ± 0.33	43.13 ± 27.38	12.32 ± 2.10
6 W	30.23 ± 0.61	43.30 ± 4.61	6.36 ± 0.70
6 WKTPP	53.07 ± 0.27	26.07 ± 3.68	2.05 ± 0.34
6 WSHMP	56.65 ± 0.65	10.35 ± 0.08	0.98 ± 0.04

CK, untreated control soil; W, wollastonite treatment; WKTPP, wollastonite with potassium triphosphate treatment; WSHMP, wollastonite with sodium hexametaphosphate treatment. Numbers before treatment group names indicate the soil Cd abundance: 0, control soil; 1, mild Cd contamination; 3, moderate Cd contamination; 6, high Cd contamination.

Zhou et al. (46) previously found that the addition of Si-containing materials to soil reduces the Cd content in wheat seeds by 16–30%. A combined application of sodium silicate and potassium dihydrogen phosphate has also been shown to reduce Cd concentrations in the roots, stems, and leaves of dicotyledonous plants by 52, 65, and 68%, respectively (17). However, other studies have reported that the application of Si-containing materials increases heavy metal accumulation in plants. For example, application of calcium silicate (CaSiO_3_) increases Cd accumulation in maize due to the growth-promoting effects of Si (47). Similarly, in kale, potassium silicate treatment increases Cd levels in the roots; potassium phosphate promotes retention of Cd in the root epidermis and endosperm of the roots and inhibits Cd transport to other tissues (17). Mao et al. (43) found that a combined W + phosphorus-containing material treatment leads to a 144% increase in Cd concentrations in brown rice compared to W treatment alone. Thus, our results were broadly consistent with those of previous studies.

Wollastonite contains Ca^2+^, which can compete with Cd^2+^ for binding sites in the roots of pak choi, thus reducing the migration of Cd to pak choi, but the addition of phosphate may disrupt this competitive adsorption and cause Ca to bind with phosphate first, so that the Cd of pak choi after the simultaneous application of wollastonite and phosphate exhibits an increase in the phenomenon shown in Figure 2. Although changes in Cd concentrations in crops after wollastonite and phosphate application were analyzed in previous studies, Cd morphology was not included. In the present study it was found that the application of compounds containing Si may also change the soil pH, thus altering the solubility of Cd^2+^ in soil water and facilitating the migration of Cd from the soil to the pak choi plant (48). Changes in the TF of soil Cd to pak choi were consistent with changes in the soil Cd fractions; i.e., increases in the more bioaccssible fractions corresponded to higher the soil Cd mobility, suggesting that differences in Cd soil fractions heavily influenced Cd transport through the soil–pak choi system. Prior studies have indicated that the total Cd mass in *Brassica napus* is related to soil heavy metal fractions. Specifically, water-soluble heavy metals have high transport capacities and bioaccessibility, whereas those in ion-exchange state are most easily absorbed and utilized by plants. Heavy metals in the carbonate-bound state are most likely to be re-released into the aqueous phase when the soil pH is altered. The addition of wollastonite and phosphate to the soil can change stable Cd to the more mobile water-soluble, ion-exchange, or carbonate-bound states, promoting accumulation in plants (Supplementary Table S2).

Overall, as wollastonite and phosphate increase the migration of Cd from soil to *Brassica napus*, and at the same time shift soil Cd to a more migratory form, the total amount of Cd in *Brassica napus* increased in most treatment groups, in which the total amount of Cd in *Brassica napus* in mildly Cd-contaminated soils was elevated by 86.2% by the treatment of WKTPP compared to the blank control, and the treatment has the potential to become a phytoremediation This treatment has the potential to be a phytoremediation aid for mildly Cd-polluted soil. The current study found that rice with low micronutrient content will result in poorer nutritional status of minerals such as calcium, iron and zinc in the human body, exacerbating the risk of Cd uptake and disease problems in the human body (49), suggesting that assessment of immobilization treatments should not focus on changes in Cd content alone. Consequently, subsequent studies should also focus on changes in the micronutrient content of pak choi to minimize and avoid compromising the nutritional value of pak choi.

### 3.3 Cd bioaccessibility in pak choi

**Figure 3:**
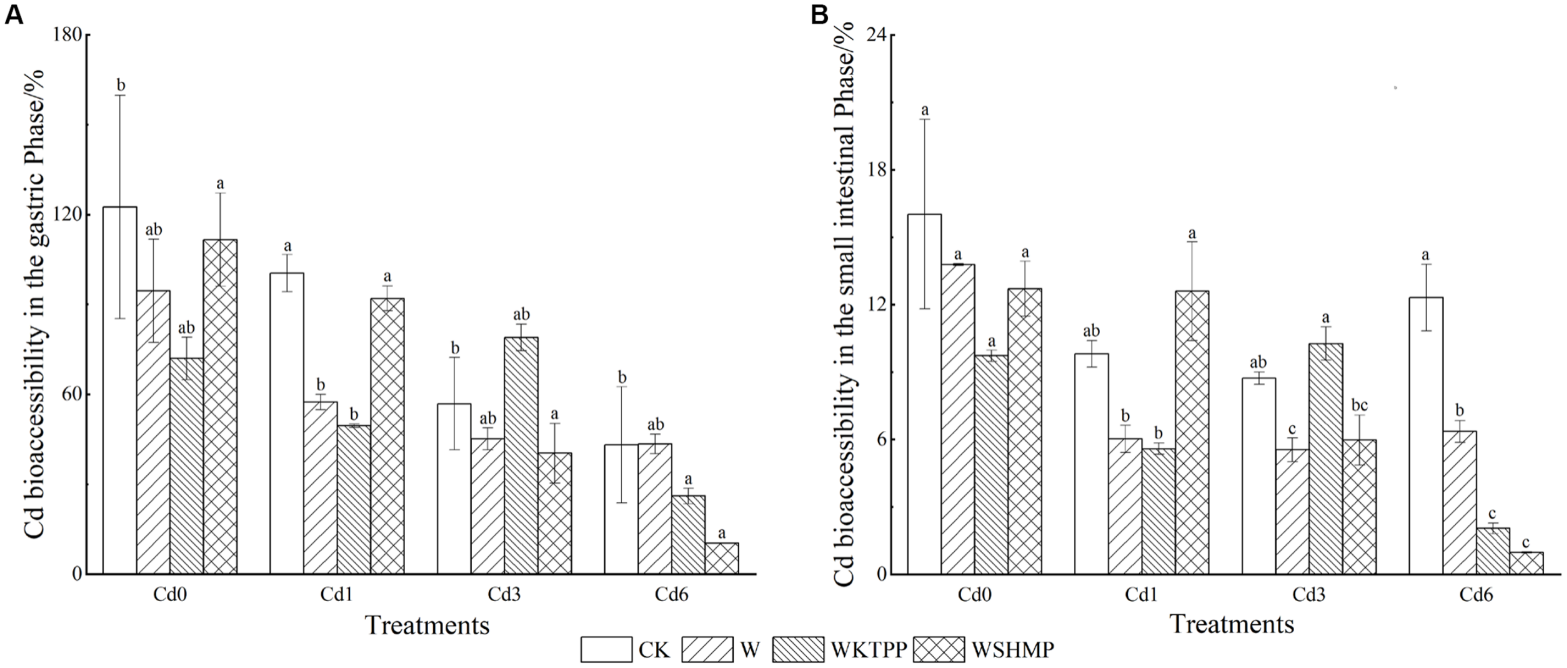
Cd bioaccessibility in pak choi grown under varying soil conditions in the **(A)** gastric and **(B)** small intestinal phases. CK, untreated control soil; W, wollastonite treatment; WKTPP, wollastonite with potassium triphosphate treatment; WSHMP, wollastonite with sodium hexametaphosphate treatment. Numbers before treatment group names indicate the soil Cd abundance: 0, control soil; 1, mild Cd contamination; 3, moderate Cd contamination; 6, high Cd contamination.

We next analyzed the gastrointestinal bioaccessibility of Cd present in pak choi using PBET. The gastric phase bioaccessibility of Cd ranged from 10.35–122.49% (mean = 65.27%) (Figure 3), which was significantly higher than Cd bioaccessibility in the small intestine (range = 0.98–16.03%; mean = 8.67%), suggesting that Cd uptake by plant tissues occurs mainly in the gastric digestive phase, which is in agreement with the findings of Qin et al. (50). This was likely due to the intensely low pH in the stomach (pH 1.5), which causes heavy metal release from plant tissues (51). Furthermore, proteases and other enzymes in the stomach break down proteins, releasing any protein-bound heavy metals (52, 53), and organic compounds in the stomach acid (such as sodium malate or sodium citrate) contain functional groups that may bind to heavy metals, increasing their accessibility and solubility (54). In contrast, the intestinal digestive environment is pH-neutral, with proteins that are inactivated in higher pH environments. Under high-pH conditions, soluble Cd often binds to digested products such as phytate, sulfur-containing amino acids, and glutathione, generating precipitates. Bile salts also reduce Cd solubility, decreasing Cd bioaccessibility in the small intestine (55).

We here found that wollastonite+phosphate treatments decreased Cd bioaccessibility in the gastric phase in both control soil and mildly Cd-contaminated soil. Compared to the untreated control, WKTPP treatment reduced Cd bioaccessibility in the gastric and intestinal phases by 50.53 and 6.3%, respectively; in mildly Cd-contaminated soils, WKTPP treatment significantly reduced Cd bioaccessibility in the gastric phase by 50.89%, whereas WSHMP increased Cd bioaccessibility in the intestinal phase by 2.78%. In moderately and highly Cd-contaminated soils, WSHMP treatment reduced gastric-phase Cd bioaccessibility by 16.49 and 32.78%, respectively. In highly Cd-contaminated soil, WKTPP treatment increased gastric-phase Cd bioaccessibility by 22.09% (Table 4).

In Figure 3 it can be seen that the addition of wollastonite and phosphate decreases the bioaccessibility of Cd in the gastric and small intestinal almost to varying degrees. This is due to the fact that Si accumulation significantly inhibit the net cellular influx of Cd^2+^ (56), whereas phosphate treatment leads to formation of Cd–phosphate complexes. These complexes adsorb to the cell wall, thus decreasing Cd transport to portions of the plant that are consumed by humans (57). Wollastonite and phosphates can mitigate heavy metal toxicity in plants and reduce bioaccessibility to in the human body through compartmentalization and structural changes that reduce metal transfer. For example, Mao et al. (43) found that wollastonite and phosphates reduce Cd bioaccessibility in rice by 29–39% by enhancing Cd incorporation into cell walls, inhibiting its transport throughout the plant. Phosphorus-containing materials (such as hydroxyapatite) cause changes in bioaccessible heavy metal fractions by altering the pH; in chili peppers and cabbages, this leads to reductions in Cd and Pb bioaccessibility of 5.0–84.8% and 5.71–93.81%, respectively (58).

The lowest bioacceesibility of Cd by WSHMP treatment was found in the study of Mao et al. This was consistent with the results in moderate and high Cd contaminated soils, but opposite to the results in mild Cd contaminated soils, which may be related to the total amount of Cd in the soil and in the pak choi (59). In the present study, Pearson’s correlation analysis showed that the total amount of Cd in pak choi was significantly negatively correlated with Cd bioaccessibility (Table 5). This suggests that high Cd concentrations in the plant were associated with a decreased capacity to release Cd from the food matrix, thus reducing Cd bioaccessibility. The addition of wollastonite and phosphate increased Si and Ca concentrations in pak choi by 50 and 10%, respectively; these increases were associated with decreased Cd bioaccessibility (Supplementary Figure S2). This is consistent with prior studies showing that CaCl_2_ treatment significantly reduces Cd bioaccessibility in rice and vegetables (34, 35). The extent of the effect of several treatments on Cd concentration differed from the effect on Cd bioaccessibility, which indicates the importance and necessity of considering the bioaccessibility of pak choi for the rational selection of fixation treatments.

**Table 5:** Correlations between Cd bioaccessibility and total Cd content in pak choi grown under varying soil conditions.

		Cd bioaccessibility from pak choi
Total Cd concentration	Pearson’s correlation	−0.789^**^
	Significance (bilateral)	<0.001
	*N*	16

^**^ Correlation is significant at the 0.01 level (2-tailed).

Generally, the bioaccessibility of Cd in pak choi was greater in the gastric phase than in the small intestine due to the influence of pH and other digestive fluids. Wollastonite and phosphate bind to and form complexes with Cd in the cell wall components of chard, and the bioaccessibility of Cd in pak choi is reduced in both cases, and the increase in the total amount of Cd in pak choi is a reduction in the ability of the food matrix to release Cd, which in turn further reduces its bioaccessibility.

### 3.4 Ultrastructural changes in pak choi due to immobilizing agent treatment

**Figure 4:**
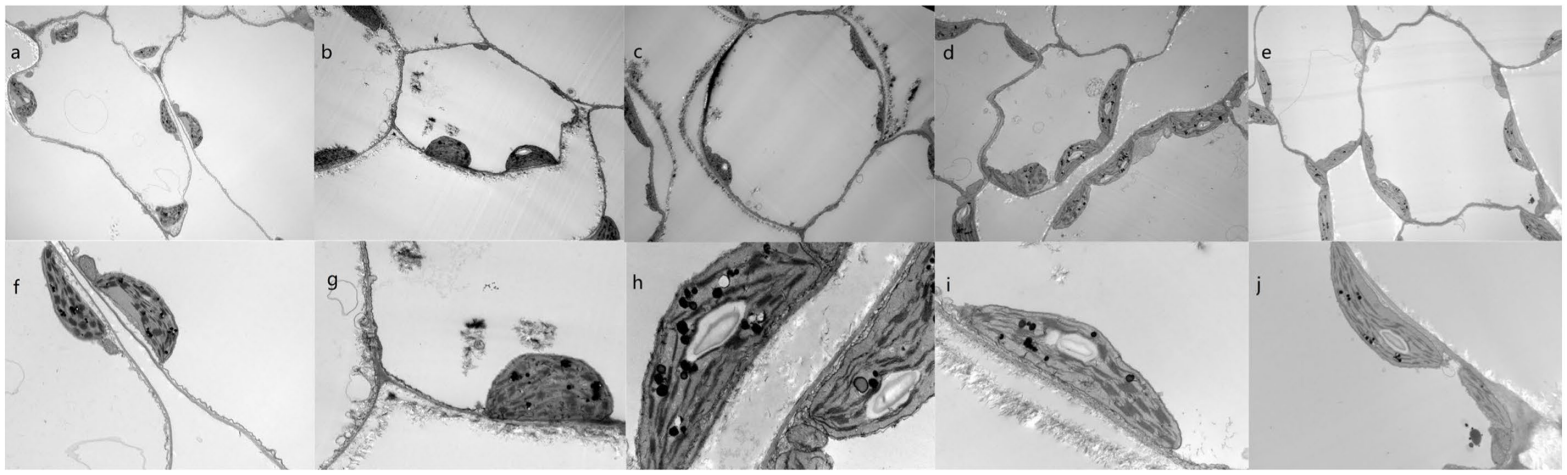
Subcellular structures of pak choi leaves from plants grown in Cd-contaminated soil with varying immobilizing treatments. **(A–J)** Cells of plants grown in the **(A,F)** blank control soil, **(B,G)** Cd-contaminated control soil, **(C,H)** Cd-contaminated soil treated with wollastonite, **(D,I)** Cd-contaminated soil treated with wollastonite and potassium triphosphate, and **(E,J)** Cd-contaminated soil treated with wollastonite and sodium hexametaphosphate. Magnificatio*n* = 5,000× **(A–E)** and 30,000× **(F–J)**.

Ultrastructural changes are often overlooked in current studies on the effects of fixation treatments on Cd in vegetables, and in order to further assess the effects of the treatments on plant morphology, we analyzed the ultrastructural characteristics of plants grown in each treatment group. No structural distortions were observed in any of the control plants (Figure 4A,F). However, the cells of leaves from plants grown in Cd-contaminated soil showed symptoms of toxicity, such as irregularly shaped cell walls and separation of cell membranes from the cell walls. Compared with plants grown in control soil, the chloroplasts of these plants were irregularly shaped; there are more osmiophilic globules increased and the granules clustered together (Figure 4B,G). Prior studies have shown that Cd exposure causes membrane lipid oxidation, significantly altering the structural features of cell membranes (60). Furthermore, cell metabolism is known to be disrupted by Cd entry into the cells, causing decreases in cell proliferation and plant biomass, which in turn increase starch granule size (61).

Here, combined applications of wollastonite and phosphate compounds also resulted in the formation of osmiophilic granules. Both these structures and the chloroplasts were of uniform size and shape, and were evenly distributed throughout the cells. These cells contained more starch granules than cells from control plants, and the cell walls and membranes were clearly defined. Impurities were isolated from the cell membranes, and the immobilizing agent treatments appeared to promote cell membrane integrity.

These ultrastructural results were consistent with earlier published findings. Heavy metals such as Cd are initially prevented from entering cells by the cell wall, which contains structural carbohydrates and proteins that act as barriers. For example, cellulose, hemicellulose, and pectin in the cell wall contain a large number of carboxyl and hydroxyl groups, which provide many binding sites for heavy metal fixation; this reduces metal solubility and decreases root-to-stem translocation (62, 63). Heavy metal contamination triggers cell wall thickening, promoting metal sequestration (64). If the heavy metal content exceeds the sequestration capacity of the cell wall, the metals are sequestered inside the cell by vesicles, which continue to protect organelles from toxicity (65).

Cd is known to interfere with chlorophyll synthase activity and to inhibit chlorophyll synthesis. Phosphate treatment of Cd-contaminated plants was here shown to cause precipitate formation (Figures 4D,E,I,J), which can reduce Cd-mediated prochlorophyllate oxidoreductase inhibition and decrease Cd-induced chlorophyll damage (66). Silicate addition also increases phosphate desorption from the soil and increases plant phosphate uptake, promoting chlorophyll synthesis.

Prior studies have been conducted to assess the effects of the subcellular heavy metal distribution within crops on heavy metal bioaccessibility. Chewing and digestion alone result in the release of heavy metal ions from leafy vegetable vesicles (67), although the release of heavy metal elements from cell walls requires cell wall protein and polysaccharide degradation by a variety of digestive enzymes (68). Wollastonite and phosphate treatments can thicken pak choi cell walls (69) and cause co-precipitation of Cd in the cell wall (47); thus, Cd is strongly sequestered in the cell wall in a non-soluble form. This prevents Cd-mediated damage to the cell membrane and vesicles; reduces Cd uptake; and reduces Cd release from pak choi into the body (Figures 4C,H). The incorporation of wollastonite and phosphate ameliorated the damage of Cd to the cellular structure of pak choi and maintained its integrity, while reducing the migration of Cd to the edible parts of pak choi, greatly reducing the health risks associated with its consumption.

### 3.5 Summary: the importance of immobilizing agents in pak choi production

Compared with traditional cereals and root crops, leafy vegetables such as pak choi are more susceptible to absorption of soil Cd. When such vegetables are consumed, Cd enters the body, which could represent a serious human health risk if the Cd has high bioaccessibility. We here sought to assess the associated risk and the effects of potential remediation strategies for soil Cd contamination. Using pak choi as a model leafy green vegetable, we here tested common immobilizing agents for soil heavy metals, including silica-containing and phosphorus-containing materials, in soils with three levels of Cd contamination. Soil Cd fractions were analyzed and pak choi cells were analyzed with transmission electron microscopy to elucidate the mechanism by which each treatment affected both the total Cd contents in pak choi and the associated bioaccessibility. These experiments yielded several key findings. First, all three treatments led to decreases in the residual soil Cd content, except for one silica–phosphorus treatment (WSHMP) in moderately Cd-contaminated soils. Furthermore, all treatments caused increases in the soil effective-state Cd concentrations, except for W treatment in moderately Cd-contaminated soil. The second key finding was that the tested treatments had varying effects on Cd bioaccessibility in pak choi plants based on the level of soil Cd contamination. Specifically, in mildly Cd-contaminated soils, WKTPP reduced Cd bioaccessibility in pak choi, whereas WSHMP had a more pronounced reducing effect on Cd bioaccessibility in pak choi in moderately and highly Cd-contaminated soils. Transmission electron microscopy observations revealed a large number of unidentified particles in the cell walls, which were inferred to be silicate complexes and insoluble precipitates formed by Cd binding with Si and P. Overall, the treatments used in this study consistently increased the total Cd content in pak choi. For example, the WKTPP treatment increased plant Cd contents by 86.2% compared to the untreated control. This finding suggests that the WKTPP treatment could be optimized for use as a phytoremediation aid for mildly Cd-contaminated soils, especially under alkaline conditions.

## 4 Conclusion

Prior evaluations of heavy metal risks to human health have focused on the total amount of a given pollutant in the environment or a specific substrate, ignoring bioaccessibility. This is a serious oversight because bioaccessibility, not total contaminant mass, ultimately determines human exposure rates. Wollastonite-conjugated phosphate immoblizingcombinations are also mostly used for remediation of Cd contamination in cereals such as rice, while Cd in leafy vegetables should be given more attention with the increasing consumption of vegetables in China. To address this issue, we here analyzed both total Cd and bioaccessible Cd in pak choi grown in Cd-contaminated soil treated with several immobilizing agents. We found that different types of silica–phosphorus treatments significantly reduced Cd bioaccessibility among pak choi plants grown in soil with varying levels of Cd contamination. Importantly, WKTPP treatment promoted Cd uptake by pak choi plants, leading to significant increases in the total Cd content in the aboveground plant tissues. At the same time, we explored the mechanisms by which wollastonite and phosphate affect Cd in pak choi at the ultrastructural level, which has often been overlooked in previous studies. These treatments could therefore play important roles in controlling the human health risks associated with Cd contamination of farmland soils; they could be used to either effectively reduce Cd bioaccessibility in crops or to maximize Cd uptake by plants optimized for soil remediation. However, *in situ* soil Cd fixation sessions had different effects on the bioaccessibility of Cd in pak choi, suggesting that there is a large uncertainty in Cd health risk assessment based simply on the total amount of Cd in pak choi. This implies that there is a need to incorporate Cd bioaccessibility into health risk assessment to accurately reflect the risk of Cd exposure from pak choi consumption. This paper provides an example of an optimal fixative treatment to reduce the bioaccessibility of pak choi. However, in order to minimize the Cd exposure risk associated with pak choi consumption, further research is needed to wind up effectively reducing Cd accumulation and bioaccessibility in pak choi. We here varied the types, but not the proportions, of silica to phosphorus immobilizing agents; future studies should address the impacts of differences in silica-containing to phosphorus-containing material ratios (and of application methods) in different soil types, and also replace the use of different combinations of silica-containing and other micronutrients. Changes in nutrients such as Zn and Se and growth-friendly enzymes should also be observed to observe more growth in pak choi. The present study provides the optimal treatment combinations that can be used for soil phytoremediation and reduction of Cd bioaccessibility in different Cd-contaminated soils in pak choi, and provides a methodology for *in-situ* soil treatments to reduce the risk of heavy metal contamination in the food chain and the environment, which promotes human and ecosystem health, and provides a theoretical basis for future environmental remediation and agri-food safety production.

## Data availability statement

The original contributions presented in the study are included in the article/Supplementary material, further inquiries can be directed to the corresponding author.

## Author contributions

KG: Conceptualization, Data curation, Methodology, Writing – original draft. YuZ: Investigation, Resources, Writing – review & editing. YaZ: Investigation, Resources, Writing – review & editing. JY: Investigation, Resources, Writing – review & editing. ZC: Investigation, Resources, Writing – review & editing. QZ: Supervision, Writing – review & editing. WX: Resources, Writing – review & editing. BH: Resources, Writing – review & editing. TL: Conceptualization, Funding acquisition, Methodology, Project administration, Writing – review & editing.
