# Supplementary material for: Effects of wollastonite and phosphate treatments on cadmium bioaccessibility in pak choi (Brassica rapa L. ssp. chinensis) grown in contaminated soils
Source: Front Nutr. 2024 Apr 4;11:1337996. doi: 10.3389/fnut.2024.1337996 (PMC11024290; doi:10.3389/fnut.2024.1337996)
Supplement: Supplementary file 1 [file Data_Sheet_1.docx]

**Effects of wollastonite and phosphate treatments on cadmium bioaccessibility in pak choi (*Brassica rapa* L. ssp. *chinensis*) grown in contaminated soils**

Kexin Guo†, Yuehua Zhao^※^, Yang Zhang†, Jinbo Yang^※^, Zhiyuan Chu^※^, Qiang Zhang†, Wenwei Xiao‡, Bin Huang§, Tianyuan Li †*

†Shandong Provincial Key Laboratory of Applied Microbiology, Ecology Institute, Qilu University of Technology (Shandong Academy of Sciences), Ji’nan, 250103, P.R. China

※The 7th Institute of Geology & Mineral Exploration of Shandong Province

† Weifang Binhai Ecological Environment Monitoring Center

‡Guangzhou Hexin Instrument Co., Ltd.

§Guoke Instrument Co., Ltd.

***List of Tables***

Table S1. Effects of insitu soil Cd immobilization on soil Cd speciation and Cd TF from soil to Pak Choi

Table S2. Correlation analysis between Total Cd concentrations of Pak Choi and Cd speciation in soil

Table S3 Two-factor ANOVA table for the effect of different treatment groups and soil Cd concentration on total Cd in Pak choi

***List of Figures***

Figure S1. Sampling locations distributed in Linyi, Shandong, China.

Figure S2. Total Ca(a) and Si(b) concentrations in pak choi after treatment with immobilizing agents.

**Table S1** Effects of insitu soil Cd immobilization on soil Cd speciation and Cd TF from soil to Pak Choi

|  | Water-  soluble  Cd  (mg kg^-1^) | Ionic bondedCd  (mg kg^-1^) | Carbonate  Cd  (mg kg^-1^) | Humic acid  bound Cd  (mg kg^-1^) | Fe/Mn  oxides  bound Cd  (mg kg^-1^) | Strong  Organic  Cd  (mg kg^-1^) | Residual  Cd  (mg kg^-1^) | TF |
| --- | --- | --- | --- | --- | --- | --- | --- | --- |
| 0CK | 0.003±  0.001 | 0.040±  0.021 | 0.032±  0.006 | 0.030±  0.011 | 0.024±  0.001 | 0.011±  0.003 | 0.043±0.01 | 1.40 |
| 0W | 0.003±  0.003 | 0.031±  0.007 | 0.032±  0.024 | 0.025±  0.013 | 0.021±  0.007 | 0.011±  0.001 | 0.037±  0.006 | 3.00 |
| 0WKTPP | 0.001±  0.001 | 0.016±  0.006 | 0.027±  0.001 | 0.016±  0.006 | 0.015±  0.003 | 0.007±  0.006 | 0.036±  0.007 | 2.65 |
| 0WSHMP | 0.001±  0.001 | 0.012±  0.001 | 0.032±  0.024 | 0.011±  0.001 | 0.006±  0.004 | 0.009±  0.006 | 0.033±  0.01 | 1.75 |
| 1CK | 0.002±  0.001 | 0.433±  0.185 | 0.361±  0.095 | 0.090±  0.021 | 0.129±  0.01 | 0.031±  0.016 | 0.041±  0.003 | 2.11 |
| 1W | 0.003±  0.001 | 0.485±  0.245 | 0.451±  0.412 | 0.124±  0.001 | 0.123±  0.042 | 0.025±  0.013 | 0.032±  0.001 | 3.06 |
| 1WKTPP | 0.001±  0.001 | 0.479±  0.115 | 0.479±  0.017 | 0.119±  0.035 | 0.115±  0.03 | 0.023±  0.001 | 0.031±  0.004 | 3.62 |
| 1WSHMP | 0.001±  0.001 | 0.481±  0.025 | 0.428±  0.311 | 0.125±  0.028 | 0.120±  0.054 | 0.023±  0.014 | 0.034±  0.004 | 2.63 |
| 3CK | 0.003±  0.003 | 0.980±  0.537 | 0.958±  0.655 | 0.225±  0.092 | 0.304±  0.216 | 0.086±  0.024 | 0.033±  0.003 | 2.43 |
| 3W | 0.004±  0.001 | 1.121±  0.218 | 0.970±  0.410 | 0.287±  0.113 | 0.401±  0.402 | 0.133±  0.001 | 0.037±  0.013 | 4.99 |
| 3WKTPP | 0.006±  0.003 | 1.208±  0.195 | 1.218±  0.495 | 0.290±  0.099 | 0.336±  0.174 | 0.079±  0.079 | 0.034±  0.003 | 4.23 |
| 3WSHMP | 0.010±  0.001 | 1.399±  0.46 | 1.485±  0.198 | 0.225±  0.049 | 0.417±  0.256 | 0.094±  0.088 | 0.057±  0.017 | 4.24 |
| 6CK | 0.012±  0.006 | 2.104±  1.482 | 1.928±  0.380 | 0.475±  0.021 | 0.687±  0.102 | 0.206±  0.225 | 0.053±  0.003 | 5.29 |
| 6W | 0.029±  0.011 | 2.636±  0.728 | 2.203±  0.313 | 0.684±  0.045 | 0.848±  0.065 | 0.199±  0.099 | 0.039±  0.007 | 4.04 |
| 6WKTPP | 0.025±  0.006 | 2.567±  0.823 | 2.120±  0.622 | 0.586±  0.32 | 0.612±  0.109 | 0.156±  0.13 | 0.040±  0.016 | 7.00 |
| 6WSHMP | 0.022±  0.004 | 2.758±  1.404 | 2.205±  1.353 | 0.500±  0.198 | 0.687±  0.102 | 0.135±  0.031 | 0.044±  0.011 | 7.99 |

CK, untreated control soil; W, wollastonite treatment; WKTPP, wollastonite with potassium triphosphate treatment; WSHMP, wollastonite with sodium hexametaphosphate treatment. Numbers before treatment group names indicate the soil Cd abundance: 0, control soil; 1, low Cd contamination; 3, moderate Cd contamination; 6, high Cd contamination.

**Table S2** Correlation analysis between Total Cd concentrations of Pak Choi and Cd speciation in soil

|  |  | Water-  soluble  Cd | Ionic bonded Cd | Carbonate  Cd | Humic acid  bound Cd | Fe/Mn  oxides  bound Cd | Strong  Organic  Cd | Residual  Cd |
| --- | --- | --- | --- | --- | --- | --- | --- | --- |
| Total Cd concentrations of Pak Choi | Pearson Correlation | 0.882^**^ | 0.945^**^ | 0.917^**^ | 0.893^**^ | 0.876^**^ | 0.817^**^ | 0.408 |
|  | Significance (bilateral) | <0.001 | <0.001 | <0.001 | <0.001 | <0.001 | <0.001 | 0.117 |
|  | N | 16 | 16 | 16 | 16 | 16 | 16 | 16 |

**Correlation is significant at the 0.01 level (2-tailed).

**Table S3 Two-factor ANOVA table for the effect of different treatment groups and soil Cd concentration on total Cd in Pak choi**

| Source of variation | Square sum | df | Mean square | F | P |
| --- | --- | --- | --- | --- | --- |
| Intercept | 11226.530 | 1 | 11226.530 | 46726.102 | ＜0.001 |
| Treatments | 578.246 | 3 | 192.749 | 802.243 | ＜0.001 |
| Soil Cd concentration | 13773.221 | 3 | 4591.074 | 19108.573 | ＜0.001 |
| Treatments* Soil Cd concentration | 1194.122 | 9 | 132.680 | 552.230 | ＜0.001 |
| Inaccuracies | 7.688 | 32 | 0.24 |  |  |

**
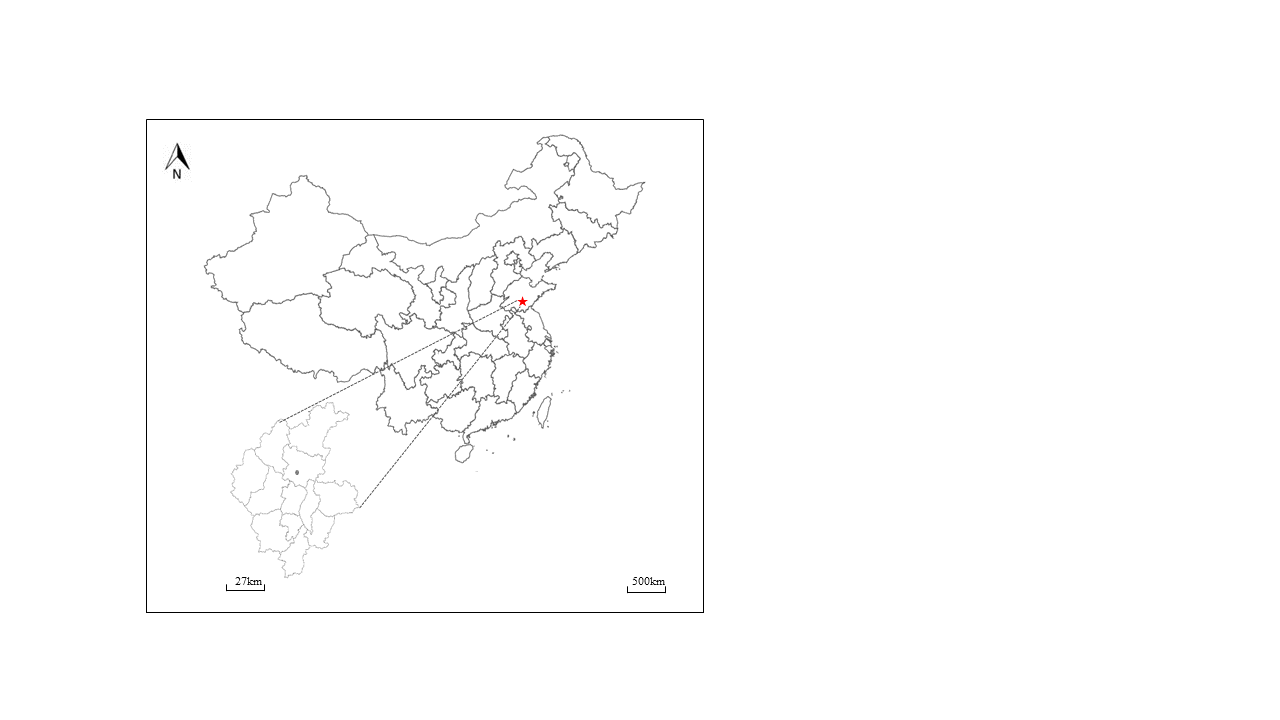
**

**Figure S1** Sampling locations distributed in Linyi, Shandong, China.


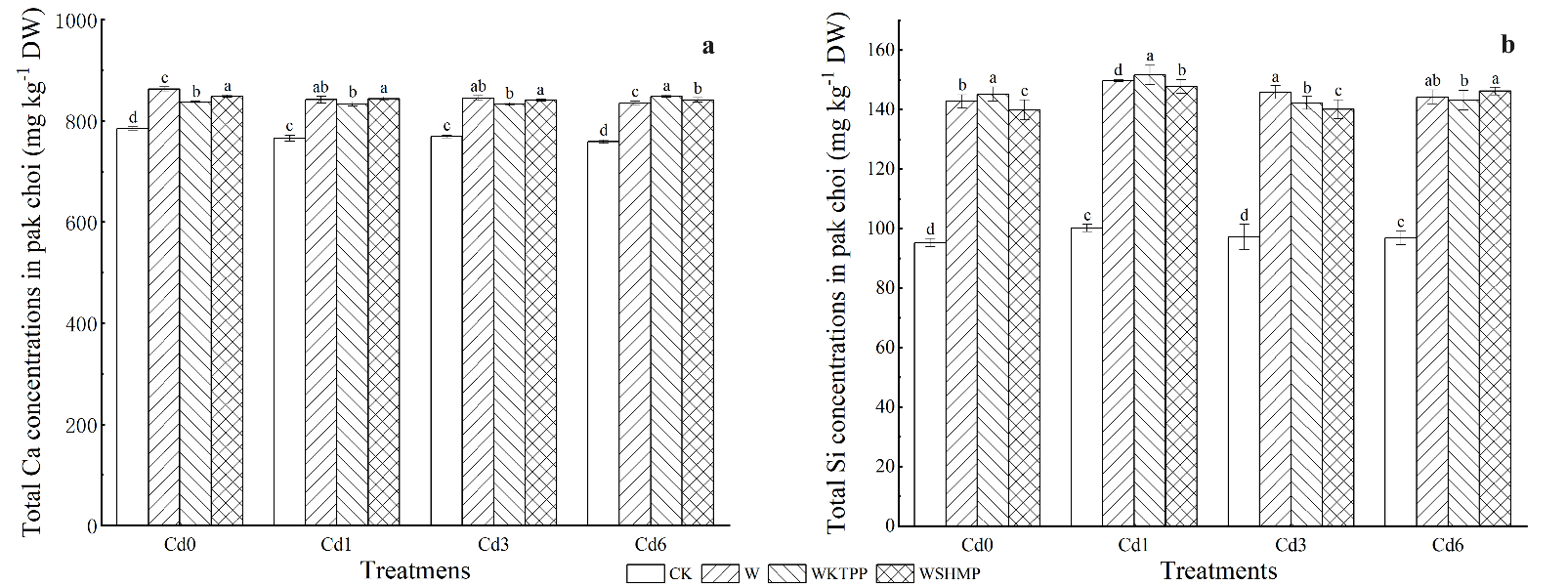


**Figure S2** Total Ca(**a**) and Si(**b**) concentrations in pak choi after treatment with immobilizing agents. CK, untreated control soil; W, wollastonite treatment; WKTPP, wollastonite with potassium triphosphate treatment; WSHMP, wollastonite with sodium hexametaphosphate treatment. Numbers before treatment group names indicate the soil Cd abundance: 0, control soil; 1, low Cd contamination; 3, moderate Cd contamination; 6, high Cd contamination. Letters above each bar indicate statistical significance groups at *p* < 0.05 (one-way analysis of variance with post-hoc Tukey’s honestly significant difference test).
